# Impact of preoperative body composition in patients with renal cell carcinoma submitted to surgical treatment

**DOI:** 10.1590/0100-3984.2023.0053

**Published:** 2023

**Authors:** Letícia Nascimento Carniatto, Almir Galvão Vieira Bitencourt, Thais Manfrinato Miola, Jéssica Agnello, Camila Machado Xavier, Walter Henriques da Costa

**Affiliations:** 1 A.C.Camargo Cancer Center, São Paulo, SP, Brazil.

**Keywords:** Carcinoma, renal cell, Body composition, Preoperative care, Computed tomography, Nutrition assessment, Carcinoma de células renais, Composição corporal, Cuidados pré-operatórios, Tomografia computadorizada, Avaliação nutricional

## Abstract

**Objective:**

To evaluate the impact of preoperative body composition in patients with renal cell carcinoma (RCC) undergoing surgical treatment.

**Materials and Methods:**

This was a retrospective study of 52 patients with RCC undergoing total or partial nephrectomy. Body composition assessment was performed using the body mass index, together with computed tomography analysis at the level of the third lumbar vertebra to measure the area of visceral adipose tissue, as well as the area and density of skeletal muscle mass.

**Results:**

Malnutrition, obesity and inadequate skeletal muscle gauge (SMG) were associated with higher hospital length of stay (*p* = 0.028, *p* = 0.02 and *p* = 0.012, respectively). Although the rates of postoperative symptoms and readmissions were low, survival was better among the patients with an adequate SMG than among those with an inadequate SMG (*p* = 0.003).

**Conclusion:**

Among patients with RCC undergoing surgical treatment, preoperative body composition does not seem to be associated with the rates of perioperative complications, although an inadequate SMG seems to be associated with worse overall survival.

## Abstract (pt)

**Objetivo:**

Avaliar o impacto da composição corporal pré-operatória em pacientes portadores de carcinoma de células renais (CCR) submetidos a tratamento cirúrgico.

**Materiais e Métodos:**

Foi realizado estudo retrospectivo de 52 pacientes portadores de CCR submetidos a tratamento cirúrgico. A avaliação da composição corporal foi realizada por meio do índice de massa corporal e análise da L3 obtida pela tomografia computadorizada para mensurar a área do tecido adiposo visceral, área e densidade da massa muscular esquelética.

**Resultados:**

Os pacientes desnutridos, obesos e que apresentaram produto muscular esquelético (PME) inadequado permaneceram mais tempo internados (p = 0,028, *p* = 0,02 e *p* = 0,012, respectivamente). As taxas de sintomas e reinternações no pósoperatório foram baixas em toda a amostra, no entanto, observou-se que pacientes com PME inadequado apresentaram uma pior sobrevida em relação aos pacientes com PME adequado (*p* = 0,003).

**Conclusão:**

A análise da composição corporal pré-operatória não mostrou associação com as taxas de complicações periope-ratórias em pacientes portadores de CCR submetidos a nefrectomia total ou parcial, no entanto, a inadequação do PME está associada a uma pior sobrevida.

## INTRODUCTION

Obesity and overweight are present in about 40–60% of cancer patients, even in those with metastatic disease, and a loss of muscle mass can be masked by excess adipose tissue^(1)^. Almost 50% of patients with early-stage renal cell carcinoma (RCC) and 29–68% of patients with advancedstage RCC have reduced muscle mass, and this is reported as the cause of death for at least 20% of patients with this type of cancer^(1,2,3)^.

Low skeletal muscle mass (SMM) is associated with the increased metabolic demand that occurs as a result of tumor malignancy, patients’ lifestyle, or malnutrition. In this process, the patients with cancer can develop low muscle strength, attenuation of muscle growth, worsening of physical performance, and in some cases, an increase in adipose tissue, leading to sarcopenic obesity^(4)^. Malnutrition and low preoperative muscle mass have been associated with worse oncological outcomes after surgery, including prolonged hospitalization, higher risks of postoperative complications, and/or higher mortality rates^(5,6,7)^.

The aim of this study was to evaluate the impact of preoperative body composition in patients with RCC undergoing surgical treatment.

## MATERIALS AND METHODS

This was a retrospective, observational, single-center study. The study was approved by the local institutional review board. Data were collected from the electronic medical records of patients diagnosed with RCC who underwent total or partial nephrectomy between January 2016 and June 2021.

The data collection included information about: anatomopathological data, presence of pre and postoperative symptoms, readmission for surgical complications within the first 30 days after discharge; oncology follow-up findings; body mass index (BMI); and preoperative computed tomography (CT) images.

**Figure 1:**
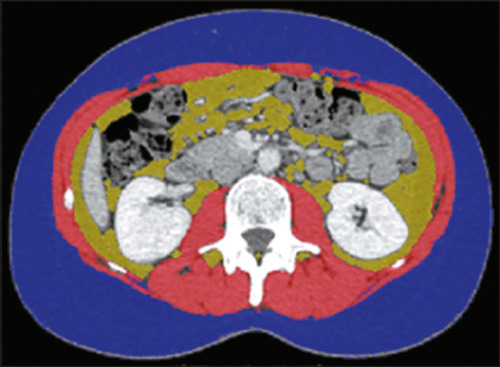
Example of a CT image at the level of the third lumbar vertebra demonstrating quantification of areas of VAT (in yellow) and SMM (in red).

The BMI was classified according to the World Health Organization criteria^(8)^ for adults and Organización Panamericana de la Salud criteria^(9)^ for the elderly. Body composition was analyzed by axial CT section at the level of the third lumbar vertebra (L3), using the CoreSlicer software (https://coreslicer.com/). To measure the areas of visceral adipose tissue (VAT) and SMM (Figure 1), we used a semiautomatic method^(10)^, with manual correction when necessary (Figure 1)^(10)^. Normal SMM was defined as tissue density between −29 HU and +150 HU, and normal VAT was defined as tissue density between -190 HU and -30 HU. A VAT between 100 cm^2^ and 130 cm^2^ was considered indicative of overweight, and a VAT > 130 cm^2^ was considered indicative of visceral obesity^(11)^. The skeletal muscle mass index (SMI) was obtained by height correction (muscle mass area in cm^2^/height in m^2^) and was considered low when it was below 52.4 cm^2^/m^2^ for men or below 38.5 cm^2^/ m^2^ for women^(5)^. Mean skeletal muscle density (SMD) was evaluated, and the cutoff points for low SMD were 35.5 HU for men and 32.5 HU for women^(6)^. In addition, the skeletal muscle gauge (SMG)–the product of the SMI and SMD–was also performed. The SMG uses muscle quantity (measured by the SMI) and muscle quality (measured by the SMD), being considered low when the value is below 1,640 and 1,523 arbitrary units for men and women, respectively^(7)^.

The data collected were stored in the using the Research Electronic Data Capture (REDCap) database (Vanderbilt University, Nashville, TN, USA). For data processing, the SPSS Statistics software package for Windows, version 17.0 (SPSS Inc., Chicago, IL, USA) was used. For the descriptive analysis of continuous variables, the measurement of central tendency (median, mean, and standard deviation) were calculated. Absolute and relative frequencies were calculated for the categorical variables. To compare the quantitative variables, Student’s t-tests or Mann-Whitney U tests were used, depending on the normality of the data distribution. To identify associations between body composition and other epidemiological, clinical and anatomopathological variables, Fisher’s exact test was used. For analysis of survival rates, we constructed Kaplan-Meier curves and used log-rank tests to compare the curves. The significance level adopted was 5%.

## RESULTS

Of the 52 patients included in the study, most (53.8%) were women. The mean age was 58.3 years. Only 10 patients (19.2%) had preoperative symptoms, among them: six (11.5%) had pain; five (9.6%) had hematuria; one (1.9%) had weight loss; and one (1.9%) had lower urinary tract symptoms. Postoperative symptoms were reported for only six patients (11.5%) and included lower urinary tract symptoms, hematuria, pain, and dehiscence of a catheter orifice (with no sign of infection). Only two patients (3.8%) were readmitted because of postoperative complications (Table 1).

**Table 1:** Clinical and demographic characteristics of patients with RCC.

Variable	(N = 52)
Sex, n (%)	
Male	24 (46.2)
Female	28 (53.8)
Age (years), mean (range)	58.3 (29–85)
Tumor type, n (%)	
Papillary	7 (13.5)
Chromophobe	5 (9.6)
Clear cell	36 (69.2)
Unclassifiable	4 (7.7)
Cancer stage, n (%)	
I or II	31 (59.6)
III or IV	19 (36.6)
No data/omitted	2 (3.8)
Preoperative symptoms, n (%)	
Yes	10 (19.2)
No	42 (80.8)
Postoperative symptoms, n (%)	
Yes	6 (11.5)
No	46 (88.5)
Readmission, n (%)	
Yes	2 (3.8)
No	50 (96.2)
Follow-up status, n (%)	
Alive and disease-free	28 (53.8)
Alive with disease	9 (17.3)
Death from cancer	8 (15.4)
Lost to follow-up	7 (13.5)

A relationship was observed between SMD and cancer stage: 75.0% of the patients with early-stage RCC had adequate SMD, compared with only 42.9% of those with advanced-stage RCC.

Regarding hospitalization, the mean length of stay (LOS) in the study population was 5.7 days (range, 0–34 days). As shown in Table 2, longer LOS was associated with malnutrition, as evaluated by the BMI, and obesity, as classified by the BMI and VAT (*p* = 0.028 and *p* = 0.02, respectively). There was no significant association between LOS and SMI (*p* = 0.709), although inadequate SMG was significantly associated with higher LOS (*p* = 0.012).

**Table 2:** Association between body composition parameters and LOS.

Variable	LOS		*P*
	Mean/median	Standard deviation	
BMI status			0.028
Underweight	10.6/2	15	
Normal weight	3.7/4	1.5	
Overweight	3/2	2.1	
Obesity	10/6	10.8	
VAT status			0.020
Adequate	5.8/4	7.09	
Overweight	2.9/2	3.1	
Obesity	6.8/4.5	8.1	
SMI status			0.709
Adequate	5.2/3	6.2	
Inadequate	6.2/3.5	8.05	
SMD status			0.158
Adequate	4.83/3	5.5	
Inadequate	6.8/4	8.8	
SMG status			0.012
Adequate	3.3/3	2.18	
Inadequate	8/4	9.3	

**Figure 2:**
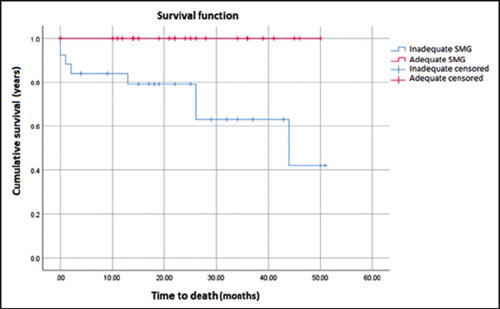
Overall survival of patients according to the SMG classification.

Among the patients evaluated, the mean survival was 47.82 months (Figure 2). Overall survival was worse in the patients with an inadequate SMG than in those with an adequate SMG (*p* = 0.003).

## DISCUSSION

The nutritional assessment of cancer patients can be performed by various methods and aims to establish early nutritional therapy^(12,13)^, helping to predict clinical and surgical outcomes^(14,15,16,17)^. In the present study, body composition was found to be associated with LOS and overall survival in patients with RCC.

In a recent study of patients with colorectal cancer, Xiao et al.^(6)^ concluded that low SMI and low SMD are associated with longer LOS, higher risks of postoperative complications, and higher mortality rates. It should also be noted that interventions in the preoperative period can help reduce complications in the postoperative period, as demonstrated by Poltronieri et al.^(17)^, who found that, in cancer patients, inadequate SMD was associated with worse clinical and surgical outcomes, such as worse overall survival and shorter progression-free survival; higher incidences of systemic inflammation and anemia; higher rates of perioperative complications; longer LOS; and toxicity of chemotherapy and radiotherapy.

Recent studies have employed the SMG in cancer patients because it measures muscle quantity and quality, although there have been only a few such studies. Young et al.^(18)^ stated that a reduction in the SMG may indicate worse survival for patients diagnosed with melanoma when it was associated with the accumulation of adipose tissue. Park et al.^(7)^ observed that, in patients with colorectal cancer, the SMG acts synergistically to improve the predictive accuracy of the SMI and SMD. In the present study, the SMG was inadequate in all of the patients who died and in most of the patients who were still undergoing cancer treatment. In addition, the patients with an inadequate SMG remained hospitalized longer and had worse overall survival.

Sharma et al.^(14)^ demonstrated that, among patients undergoing cytoreductive nephrectomy, those with low SMM had a lower BMI, were more likely to have hypoalbuminemia before surgery, required more blood transfusions in the perioperative period, remained hospitalized for longer, and had shorter overall survival. Emphasizing that malnutrition and loss of muscle mass are known to be common in and to have a negative effect on the clinical evolution of patients with cancer^(12)^, as was corroborated by the results obtained in the present study.

The distribution and quantity of adipose tissue have been associated with postoperative complications in patients undergoing minimally invasive partial nephrectomy^(19)^. However, postoperative complication rates may be associated with the surgical technique, given that laparoscopic radical nephrectomy is associated with less blood loss and a shorter recovery period in comparison with open radical nephrectomy^(20)^. In the present study, 59.6% of the patients were diagnosed with RCC in the early stages and the surgical technique used in most cases was laparoscopic nephrectomy, which may explain the low rates of postoperative symptoms and readmissions (11.5% and 3.8%, respectively). In addition, all of the patients who presented postoperative symptoms were classified as overweight or obese on the basis of the VAT.

Our study has some limitations. First, it was a retrospective study, with all of the biases inherent to that design. In addition, the number of patients evaluated was small because of the unavailability of CT images of the abdomen. Despite those limitations, we have demonstrated that the early assessment of body composition can complement the prediction of clinical outcomes in patients with RCC. We emphasize the high prevalence of low SMM and excess body weight in our study population, which underscores the importance of using accurate tools to assess body composition throughout the follow-up period in order to implement individualized nutritional interventions. It is also noteworthy that the various tools employed to assess nutritional status can be used simultaneously, providing the data required to make a more accurate nutritional diagnosis.

## CONCLUSION

Among patients with RCC undergoing total or partial nephrectomy, preoperative body composition does not seem to be associated with the rates of perioperative complications, although inadequate SMG seems to be associated with worse overall survival. In our sample of such patients, LOS was significantly associated with BMI, VAT, and the SMG.
